# Three-Dimensional Subject-Specific Knee Shape Reconstruction with Asynchronous Fluoroscopy Images Using Statistical Shape Modeling

**DOI:** 10.3389/fbioe.2021.736420

**Published:** 2021-10-20

**Authors:** Hsuan-Yu Lu, Kao-Shang Shih, Cheng-Chung Lin, Tung-Wu Lu, Song-Ying Li, Hsin-Wen Kuo, Horng-Chaung Hsu

**Affiliations:** ^1^ Department of Biomedical Engineering, National Taiwan University, Taipei, Taiwan; ^2^ Department of Orthopedics, Shin Kong Wu Ho-Su Memorial Hospital, Taipei, Taiwan; ^3^ School of Medicine, Fu Jen Catholic University, Taipei, Taiwan; ^4^ Department of Electrical Engineering, Fu Jen Catholic University, Taipei, Taiwan; ^5^ Department of Orthopaedic Surgery, School of Medicine, National Taiwan University, Taipei, Taiwan; ^6^ Department of Orthopaedic Surgery, China Medical University, Taipei, Taiwan

**Keywords:** statistical shape model, subject-specific, knee joint, digitally reconstructed radiographs, two-phase optimization

## Abstract

**Background and objectives:** Statistical shape modeling (SSM) based on computerized tomography (CT) datasets has enabled reasonably accurate reconstructions of subject-specific 3D bone morphology from one or two synchronous radiographs for clinical applications. Increasing the number of radiographic images may increase the reconstruction accuracy, but errors related to the temporal and spatial asynchronization of clinical alternating bi-plane fluoroscopy may also increase. The current study aimed to develop a new approach for subject-specific 3D knee shape reconstruction from multiple asynchronous fluoroscopy images from 2, 4, and 6 X-ray detector views using a CT-based SSM model; and to determine the optimum number of planar images for best accuracy via computer simulations and *in vivo* experiments.

**Methods:** A CT-based SSM model of the knee was established from 60 training models in a healthy young Chinese male population. A new two-phase optimization approach for 3D subject-specific model reconstruction from multiple asynchronous clinical fluoroscopy images using the SSM was developed, and its performance was evaluated *via* computer simulation and *in vivo* experiments using one, two and three image pairs from an alternating bi-plane fluoroscope.

**Results:** The computer simulation showed that subject-specific 3D shape reconstruction using three image pairs had the best accuracy with RMSE of 0.52 ± 0.09 and 0.63 ± 0.085 mm for the femur and tibia, respectively. The corresponding values for the *in vivo* study were 0.64 ± 0.084 and 0.69 ± 0.069 mm, respectively, which was significantly better than those using one image pair (0.81 ± 0.126 and 0.83 ± 0.108 mm). No significant differences existed between using two and three image pairs.

**Conclusion:** A new two-phase optimization approach was developed for SSM-based 3D subject-specific knee model reconstructions using more than one asynchronous fluoroscopy image pair from widely available alternating bi-plane fluoroscopy systems in clinical settings. A CT-based SSM model of the knee was also developed for a healthy young Chinese male population. The new approach was found to have high mode reconstruction accuracy, and those for both two and three image pairs were much better than for a single image pair. Thus, two image pairs may be used when considering computational costs and radiation dosage. The new approach will be useful for generating patient-specific knee models for clinical applications using multiple asynchronous images from alternating bi-plane fluoroscopy widely available in clinical settings. The current SSM model will serve as a basis for further inclusion of training models with a wider range of sizes and morphological features for broader applications.

## Introduction

The knee joint plays an essential role in the normal function of the lower extremities, providing stability and mobility necessary for locomotion while bearing the body’s weight. Knowledge of the biomechanical interactions of the bones and their surrounding force-bearing tissues is thus crucial for a better understanding of the functional changes of the knee under normal, pathological and post-surgical conditions (Wilson et al., 1998; Eck et al., 2002; Kleipool and Blankevoort, 2010). To this end, it is critical to be able to describe accurately individual-specific morphology of bones. These descriptions play important roles in various clinical applications such as fluoroscopy-based kinematic measurement (Lu et al., 2008), pre-surgical planning (Krekel et al., 2006), customized finite element analysis (Fernandez et al., 2004), joint implant design (Cheng et al., 1999; Harrysson et al., 2007) and computer-aided orthopaedic surgery (Gamage et al., 2009).

An accurate description of the shapes of bones is particularly important in bone model-based tracking techniques for *in vivo* measurement of three-dimensional (3D) joint kinematics for evaluating functional changes owing to disorders and/or treatments (Georgoulis et al., 2003; McDonald et al., 2014; Kobayashi et al., 2015), and for deriving soft tissue deformations and forces (Li et al., 2004; Haraguchi et al., 2009; Wang et al., 2013; Ding and Khan, 2019). Among the existing methods, fluoroscopy-to-CT or MRI registration techniques have been shown to be effective and less-invasive in measuring the 3D kinematics of various joints during weight-bearing functional activities (Li et al., 2008; Anderst et al., 2009; Tsai et al., 2013; Lin et al., 2014a). These techniques use subject-specific, CT- or MRI-based bone models and determine their 3D pose by searching for the final pose of the bone model whose digitally reconstructed radiograph or projections on the image plane best match the fluoroscopy image(s) (Tsai et al., 2010). Techniques using single-plane fluoroscopy have been applied to 3-D kinematic measurements of normal (Lu et al., 2008; Yamaguchi et al., 2009), pathological (Dennis et al., 2005; Hamai et al., 2009; Kobayashi et al., 2015) and replaced joints (Dennis et al., 2003; Liu et al., 2007; Yamaguchi et al., 2011), but the measured translations normal to the image plane are substantially less accurate than those of the other components (Fregly et al., 2005; Lin et al., 2014b). Bi-plane approaches address the issue by simultaneously incorporating an additional X-ray image plane, achieving much higher accuracy (Li et al., 2008; Anderst et al., 2009; Brainerd et al., 2010; Tsai et al., 2013; Lin et al., 2014b; Kapron et al., 2014; Ito et al., 2015). Generally, CT-based models are more accurate than MRI-based models but the radiation exposure is of concern. Therefore, reconstruction of three-dimensional patient-specific bone models of accuracy comparable to that of CT-based models from planar radiographs or fluoroscopy images will be useful for reducing the radiation exposure.

Statistical shape modeling (SSM) techniques have been used in the development of fully automated bone segmentation methods (Lamecker et al., 2004; Josephson et al., 2005; Fripp et al., 2006), parametric descriptions of the bony geometry (Seber, 2009) and reconstruction of subject-specific bone models using 2D/3D registration (Baka et al., 2011; Valenti et al., 2016a; Valenti et al., 2016b; van IJsseldijk et al., 2016; Yu et al., 2016; Cerveri et al., 2017; Smoger et al., 2017; Yu et al., 2017; Zheng and Yu, 2017; Fotsin et al., 2019; Cerveri et al., 2020; Wu and Mahfouz, 2021). With the SSM, a bone model is described as the mean shape superimposed by a linear combination of the principal components of shape variations of the training dataset. Reconstruction of a bone model thus involves determining the coefficients of the linear combination that best match subject-specific information such as from one or more radiographs (Lamecker et al., 2006; Baka et al., 2011; Sarkalkan et al., 2014; Karade and Ravi, 2015). So far, existing SSM models are built based mainly on Caucasian populations (Baka et al., 2011; Baka et al., 2012; Karade and Ravi, 2015; Tsai et al., 2015). However, morphological differences have been noted between Chinese and Caucasian population (Cheng et al., 1999; Mahfouz et al., 2012). For example, Cheng *et al* showed significant differences in the ratios of anteroposterior and mediolateral dimensions between the resected surfaces of the tibial plateau in a Chinese patient group and the tibial component of a total knee replacement designed based on the Caucasian population (Cheng et al., 1999). They suggested that between-population differences in the knee morphology directly impact the design and implantation of total knee replacements. Another study by Mahfouz et al. (2012) also identified significant differences in the mean dimensions of the three-dimensional morphology of the distal femur and proximal tibia among different ethnic populations. These previous results show that knee shape variations among multiple ethnic groups are greater than those of a single ethnic group. Therefore, an SSM dataset aiming to address more than one ethnic group would need a sample size greater than that for a single ethnic group. Considering the challenge for a dataset big enough to cover the shape variations in multiple ethnic groups and the gap in the SSM for the Chinese population, there is a need to establish a CT-based SSM for a Chinese group, which can be further expanded to include a wider range of subjects for basic research and clinical applications.

The accuracy of the model’s reconstruction using SSM can be affected by the number of radiographic images and the algorithm used. Theoretically, the more the images used, the more accurate the reconstructed bone model. Alternating bi-plane fluoroscopy systems are widely available in hospitals, providing a single pair of asynchronous fluoroscopy images. More image pairs can be obtained but movement of either the bones or the clinical fluoroscopy systems cannot be avoided, leading to temporally and spatially asynchronous images. Previous studies have mainly used a single fluoroscopy image (Fleute and Lavallée, 1999; Lamecker et al., 2006; Zheng et al., 2006; Wu and Mahfouz, 2021) or a single pair of fluoroscopy images (Laporte et al., 2003; Tang and Ellis, 2005; Baka et al., 2011; Zhu and Li, 2011; Baka et al., 2012) for 3D model reconstruction. More recent computer simulation studies used multiple image pairs for model reconstruction but considered only synchronous images (Valenti et al., 2016a; Valenti et al., 2016b; Smoger et al., 2017), so the methods proposed in these studies cannot be applied to alternating bi-plane fluoroscopy systems in clinical settings. Moreover, the accuracies reported were not representative of those in the scenarios of clinical applications. Up to the present, no study was found to evaluate the effects of image number on the accuracy of SSM-reconstructed personalized bone models of the knee to determine systematically the optimum number of planar images for personalized SSM-model reconstruction via an *in vivo* experimental setup.

The current study aimed to develop a new approach for 3D subject-specific knee shape reconstruction from multiple asynchronous fluoroscopy images from 2, 4, and 6 X-ray detector views using a CT-based SSM model of the knee; and to determine the optimum number of planar images for the new approach by systematically evaluating the effects of image number on reconstruction accuracy via computer simulation and *in vivo* data. It was hoped that the new approach would be used not only with the current SSM model but also with other existing SSM models for 3D subject-specific knee shape reconstruction from asynchronous fluoroscopy images, and that the current CT-based SSM model could form a basis for further inclusion of a wider range of subjects for basic research and clinical applications.

## Materials and Methods

### Statistical Shape Modeling of the Knee

**FIGURE 1:**
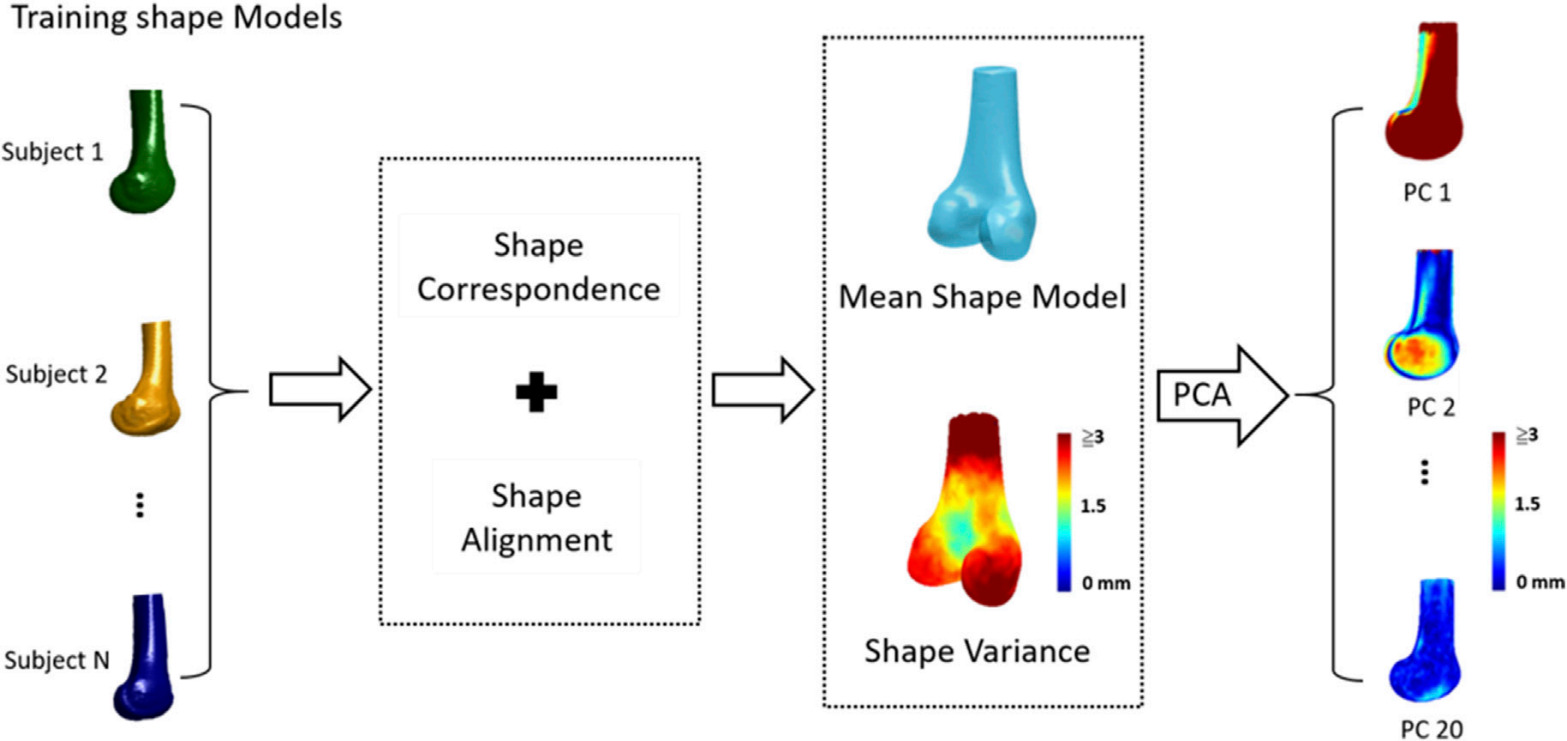
The general procedure of statistical shape modeling (SSM) for the human knee: 1) a set of CT-derived training shape models, 2) choosing a reference model; 3) establishing shape (mesh) correspondence and alignment between individual training models; 4) determining the mean shape model and shape variations; and 5) determining primary modes of shape variations using Principal Component Analysis (PCA).

The general procedure of the SSM of the knee included 1) obtaining a set of CT-derived training shape models, 2) choosing a reference model with a predefined surface mesh; 3) establishing shape (mesh) correspondence between individual training models by transforming the reference model to individual training ones; and 4) determining the mean model and primary modes of shape variations using Principal Component Analysis (PCA) (Figure 1).

#### Training Shape Models of the Knee

The training shape models of the knee were reconstructed from the CT data of the distal femur and the proximal tibia from 60 healthy Chinese males (age: 22.89 ± 2 years; body height: 172.64 ± 5.2 cm; body mass: 66.35 ± 10.6 kg) who gave informed written consent as approved by the Institutional Review Board. The CT datasets were acquired for a total length of about 420 mm with a voxel size ranging from 0.441 mm × 0.441 mm × 0.625–0.822 mm × 0.822 mm × 0.625 mm (PQ-5000, Picker International, United States). The regions of the femur and tibia were segmented semi-automatically and reconstructed to obtain subject-specific training shape models using ITK-SNAP 3.6.0 (University of Pennsylvania, United States).

#### Shape Correspondence

Shape correspondence between individual training shape models was established by applying a self-organizing network with the Growing and Adaptive Meshes (GAMEs) algorithm (Ferrarini et al., 2007) to a randomly-selected knee model. A reference model was then selected as the model that was closest to the mean shape of the individual models. The reference model was spatially aligned to each of the training shape models via the Iterative Closet Point (ICP) method (Besl and McKay, 1992), and subsequently deformed non-rigidly to match fully with the shape model using the Coherent Point Drift (CPD) method (Myronenko and Song, 2010), yielding training shape models with corresponding meshes. The CPD method has been proven to have robust and accurate performance with respect to noise, outliers and missing points (Myronenko and Song, 2010).

#### Shape Alignment and Shape Variation

All the training models with corresponding meshes were best-aligned using Generalized Procrustes Analysis (GPA), minimizing the surface distances between all the models (Goodall, 1991; Cootes and Taylor, 2004). These best-aligned models were then analyzed using Principal Component Analysis (PCA) (Wold et al., 1987) to give a set of eigenvalues $\lambda_{i}$ and eigenvectors $\phi_{i}$ , with which the form of the SSM was given as follows.

$$m_{s}=\bar{m}+\overset{c}{\underset{i=1}{\sum }}b_{i}\phi_{i}$$ (1)

where $m_{s}$ is a training shape model; $\bar{m}$ is the mean shape of all training models; $\phi_{i}$ indicates the principal modes of the shape variation following the descending order of the corresponding eigenvalues $\lambda_{i}$ ; $b_{i}$ are shape parameters bounded within an interval of $\left[-3\sqrt{\lambda_{i}}\;,3\sqrt{\lambda_{i}}]\right]$ (Cootes and Taylor, 2004; Sarkalkan et al., 2014); and *c* is the number of principal modes used for generating $m_{s}$ such that the ratio of the accumulated variance of eigenvalues to the total variance reaches a given level such as 0.9–0.98 (Heimann and Meinzer, 2009).

### Reconstruction of Personalized Statistical Shape Model

**FIGURE 2:**
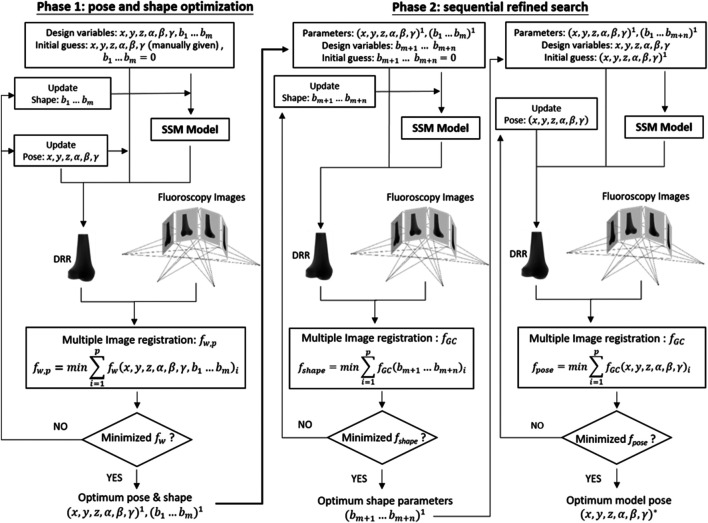
Two-phase optimization scheme for reconstructing a personalized statistical shape model of the knee. In the current study, *m* = 10 and *n* = 10 were taken.

To generate personalized bone shape model using the trained SSM with asynchronous 2D images of the bone, a two-phase optimization scheme was developed as described below (Figure 2).

#### Digitally Reconstructed Radiograph

The two-phase optimization scheme utilized a 2D fluoroscopy to 3D model registration technique. This required the generation of digitally reconstructed radiographs (DRR) of the volumetric model of the bone onto the image planes of the fluoroscopy system. Generally, the planar detector of the fluoroscopy system was modeled as a perspective projection system, and the parameters for the positions of the X-ray sources relative to the image plane and possible image distortions were obtained via a validated calibration procedure (Tsai, 1987; Baltzopoulos, 1995; Kaptein et al., 2011). Under the perspective projection model, DRR of the bone was then produced using the ray-tracing method with trilinear interpolation (Otake et al., 2011), casting rays from a point source of x-ray to each pixel position of an image plane through the volumetric bone model. Each of these rays went through a number of voxels of the volume, the linear attenuation coefficient values of which were then integrated along the ray and projected onto the imaging plane to obtain a DRR image resembling a radiograph (Siddon, 1985; Penney et al., 2001; De Bruin et al., 2008).

#### Phase 1: Pose and Shape Optimization

The first phase of the new method involved the search for the optimum set of design variables to maximize the similarity between the DRRs of the volumetric bone model and the multiple asynchronous 2D fluoroscopy images (Figure 2). The design variables were the pose parameters (i.e., six degrees-of-freedom of the bone) and the coefficients for the first *m* principal modes of the shape variation, i.e., *m* shape parameters $b_{i},\;i=1,m$ . The initial guesses of the pose parameters of the mean shape models of the femur and tibia were manually assigned separately by an operator. For generating the DRRs of the volumetric bone model, the shape model defined by the shape parameters was voxelized to simulate the CT-based bone model by filling the interior of the shape model with voxels by taking intersections of the interior of the shape model, and a set of parallel virtual 2-D slices in the transverse plane with a pixel size of 1 × 1 mm and slice thickness of 1 mm (Patil and Ravi, 2005). The resulting virtual voxels interior to the shape model were assigned a value of 700 to simulate the Hounsfield unit (HU) value of bone, while voxels outside the contours were assigned −1,000 to simulate air.

For a given set of design variables, the DRRs of the voxelized bone model in space were generated and compared with each corresponding fluoroscopy image according to the a similarity measure called Weighted Edge-Matching Score (WEMS, $f_{w}$ ) that emphasized the alignment of longer edges between the DRRs and fluoroscopy images (Tsai et al., 2010). For the *i*th fluoroscopy image, the edges of the image ( $E_{f,i}$ ) were first detected using the Canny operator (Canny, 1986), and then dilated with a given band to give $B_{f,i}$ . Similarly, the edges of the DRR were also detected to give $E_{DRR,i}$ . The separated edges in $\;E_{DRR,i}$ were given weightings depending on their lengths and stored in a weighting image $W_{DRR,i}$ . The *WEMS* values to be minimized were defined as follows.

$$f_{w}{\left(\text{x},\text{y},\text{z},\text{α},\text{β},\text{γ},b_{1},\ldots ,b_{m}\right)}_{i}=\frac{-\sum_{\left(x,y\right)}B_{f,i}\left(x,y\right)\cdot W_{DRR,i}\left(x,y\right)}{\sum_{\left(x,y\right)}W_{DRR,i}\left(x,y\right)}$$ (2)

For *p* fluoroscopic images, the combined similarity measure to be minimized is defined as follows.

$$f_{w,p}=\;\overset{p}{\underset{i=1}{\sum }}f_{w}{\left(x,y,z,\alpha ,\beta ,\gamma ,b_{1},\ldots ,b_{m}\right)}_{i}$$ (3)

The initial shape parameters $b_{1},\ldots ,b_{m}$ were set to be zero because the mean training model and initial pose parameters were manually given via a graphic user interface. The resulting optimization problem was solved using a genetic algorithm (Goldberg and Holland, 1988).

#### Phase 2: Sequential Refined Search

In the second phase, the shape model and its pose obtained in Phase 1 were refined further in two steps. At the first step, the shape model was further refined by including *n* additional shape parameters, i.e., $b_{m+1},\ldots ,b_{m+n}$ , for a better match with *p* fluoroscopy images taking the pose and shape parameters obtained in Phase 1 as fixed parameters. The similarity between the model-projected DRRs and the corresponding fluoroscopy images for this refining search was defined using a metric called the gradient correlation (GC, $f_{GC}$ ) (Penney et al., 1998). The sum of the $-f_{GC}$ of *p* fluoroscopy images was taken as the cost function to be minimized, as follows.

$$f_{shape}=\;\overset{p}{\underset{i=1}{\sum }}-f_{GC}{\left(b_{m+1},\;\ldots ,b_{m+n}\right)}_{i}$$ (4)

The resulting optimization problem was solved using a genetic algorithm (Goldberg and Holland, 1988), giving the final shape of the bone model described by $b_{1},\ldots ,b_{m+n}$ . At the second step with the final bone model, the pose parameters were further refined such that the model-projected DRRs best matched the *p* fluoroscopy images, minimizing the sum of the $-f_{GC}$ of *p* fluoroscopy images as follows.

$$f_{pose}=\;\overset{p}{\underset{i=1}{\sum }}-f_{GC}{\left(x,y,z,\alpha ,\beta ,\gamma \right)}_{i}$$ (5)

The resulting optimization problem was also solved using a genetic algorithm (Goldberg and Holland, 1988), giving the final pose of the bone model.

### Evaluation of the SSM

The performance of the proposed SSM method was evaluated using computer simulation and *in vivo* studies as described below.

#### Computer Simulation

**FIGURE 3:**
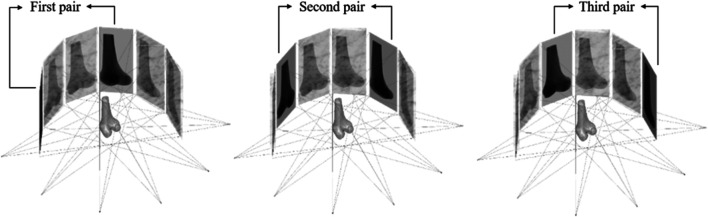
Illustration of the views of simulated X-ray images for three image pair conditions. The first image pair was obtained from the starting reference detector position. The second and third image pairs were obtained by rotating the bi-plane fluoroscope around the isocenter from the reference detector position by 30° and 60°, respectively.

Computer simulations using a leave-one-out cross-validation scheme were performed to evaluate the proposed SSM and personalized reconstruction procedure under ideal conditions using synchronized images. Six out of the 60 subjects from the training shape model were randomly chosen one at a time, and the corresponding CT-based model was used to simulate the personalized reconstruction procedure, but was ruled out in building the SSM. For each selected subject, multiple X-ray image pairs simulating those produced from the bi-plane fluoroscopy system were generated by perspective projection of the CT-based volumetric knee model onto the image planes of the simulated fluoroscope. The CT-based volumetric knee model was positioned at the isocenter of the simulated fluoroscopy system, and three combinations of asynchronous X-ray image pairs (DRR pairs) were produced for evaluating the personalized reconstruction process: 1) one image pair (two orthogonal images) simulating the bi-plane fluoroscopy system; 2) two image pairs (four images produced by rotating the simulated bi-plane fluoroscope around the isocenter by 45°); 3) three image pairs (six images produced by rotating the simulated bi-plane fluoroscope around the isocenter by 30° and 60°) (Figure 3). The 3D personalized model of the knee was reconstructed from each of the three combinations of asynchronous image pairs using the proposed reconstruction procedure.

#### *In vivo* Study

Ten healthy young male volunteers (age: 23 ± 2.36 years; height: 174.6 ± 4.27 cm; body mass: 63.6 ± 9.92 kg) without any neuromusculoskeletal disease or any surgical history of the lower limbs participated in the current study. The subjects were fully informed of the experimental protocol as approved by the Research Ethics Committee of China Medical University and Hospital (approval number: CMUH107-REC2-078), and gave their written consent prior to the experiment. All the subjects were scanned by CT (PQ-5000, Picker International, United States) to reconstruct the volumetric model of the knee with a voxel size of 0.709 × 0.709 × 0.625 mm. A bi-plane fluoroscopy system (Allura XPER FD 20/20, Philips Medical Systems, Netherlands) was used to acquire the dynamic X-ray images at 512 × 512 resolution during the experiment. Prior to data acquisition, intrinsic and extrinsic parameters for the bi-plane projection models of the fluoroscopy system were determined following well-established calibration procedures (Kaptein et al., 2011). Four lead markers were attached on the distal thigh and proximal shank, and one lead marker was attached on the patella. The subject was then asked to stand on a rotating plate with the knee located at the isocenter of the bi-plane imaging system. Using the plate, the lower limb was rotated sequentially by 0°, 30°, 45°, and 60° about the vertical axis while the bi-plane X-ray images were acquired. The 3D coordinates of the lead markers were determined using radiostereometric analysis for each pair of fluoroscopy images (Karrholm, 1989). Transformations between the X-ray image pairs were obtained by co-registering the known coordinates of the lead markers, from which the three combinations of image pairs were obtained as in the computer simulation study.

#### Error Metrics

To evaluate the performance of the proposed SSM and subject-specific model reconstruction procedure using different numbers of image pairs, the shape differences between the reconstructed model and the corresponding gold-standard CT-based model were quantified using a metric based on point-to-surface distances as follows (Cignoni et al., 1998):

$$\text{e}\left(\text{p},\text{S}\right)=\;\underset{p^{\prime }\epsilon S}{\min}d\left(p,p^{\prime }\right)$$ (6)

For each point p on the surface of the SSM-generated personalized bone model, its shortest Euclidean distance e to a point $p^{\prime }$ on the surface S of the CT-derived bone model was calculated, and the root-mean-squared values of e over the surface points (RMSE) was then obtained as a measure of the performance of the proposed method.

### Statistical Analysis

Means and standard deviations of the RMSE were obtained for both computer simulation and *in vivo* study. For computer simulations, univariate analysis with a polynomial linear test was performed to determine the trend of the reconstruction accuracy variables with increasing image pairs. For the *in vivo* study, paired *t*-tests were used to compare the accuracy between image pair conditions, and between the results of Phases 1 and 2 of the two-phase optimization method. All statistical analyses were performed using SPSS (SPSS Inc., Chicago, United States) at a significance level set at 0.05.

## Results

### Principal Component Analysis

**FIGURE 4:**
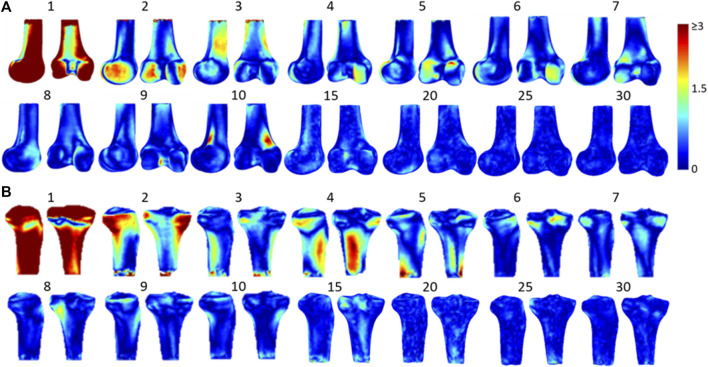
The 3D shape variation of SSMs models of **(A)** the femur and **(B)** the tibia with colors representing the difference caused by each principal component on the reference model. The first few principal components account for most of the total shape variance.

From the principal component analysis of the training models, it was found that the first principal component of the femur and tibia accounted for 70.7 and 58.9% of the total shape variance, respectively, while the accumulated variance of the first ten, first twenty and first thirty consecutive principal components were 85.32, 91.13, and 94.91% for the femur respectively, and 84.14, 90.45, and 94.86% for the tibia, respectively. For the femur, the first principal component was related to the deformation of the overall shape of the femur, while the second one was related to the deformation of the medial and lateral condyles, and the third one to the medial and lateral aspects of the femoral shaft (Figure 4). For the tibia, the first principal component would deform the overall shape of the tibia while the second one would deform the medial and lateral condyles and the radius of the shaft, and the third one was related to the variance in the shapes of the shaft of the tibia (Figure 4).

### Computer Simulation

The RMSE of the reconstructed knee models using one image pair for the femur and tibia were 0.62 ± 0.075 and 0.72 ± 0.076 mm, respectively. The corresponding values for two and three image pairs were 0.57 ± 0.088 and 0.67 ± 0.074 mm, and 0.52 ± 0.09 and 0.63 ± 0.085 mm, respectively, (Table 1). The RMSE of the reconstructed knee models decreased linearly as the number of fluoroscopy image pairs increased.

**TABLE 1:** The means (standard deviations) of the RMSE (unit: mm) for the femur and tibia shape reconstruction using 1, 2, and 3 image pairs by computer simulation. *p* values of a univariate analysis with a polynomial linear test are also given for the trend of reconstruction accuracy with increasing image pairs.

		1 image pair	2 image pairs	3 image pairs	p
Computer simulation	Femur	0.62 (0.075)	0.57 (0.088)	0.52 (0.09)	0.048^*^
	Tibia	0.72 (0.076)	0.67 (0.074)	0.63 (0.085)	0.049^*^

*: *p*< 0.05, significant linear trend.

### *In vivo* Study

**FIGURE 5:**
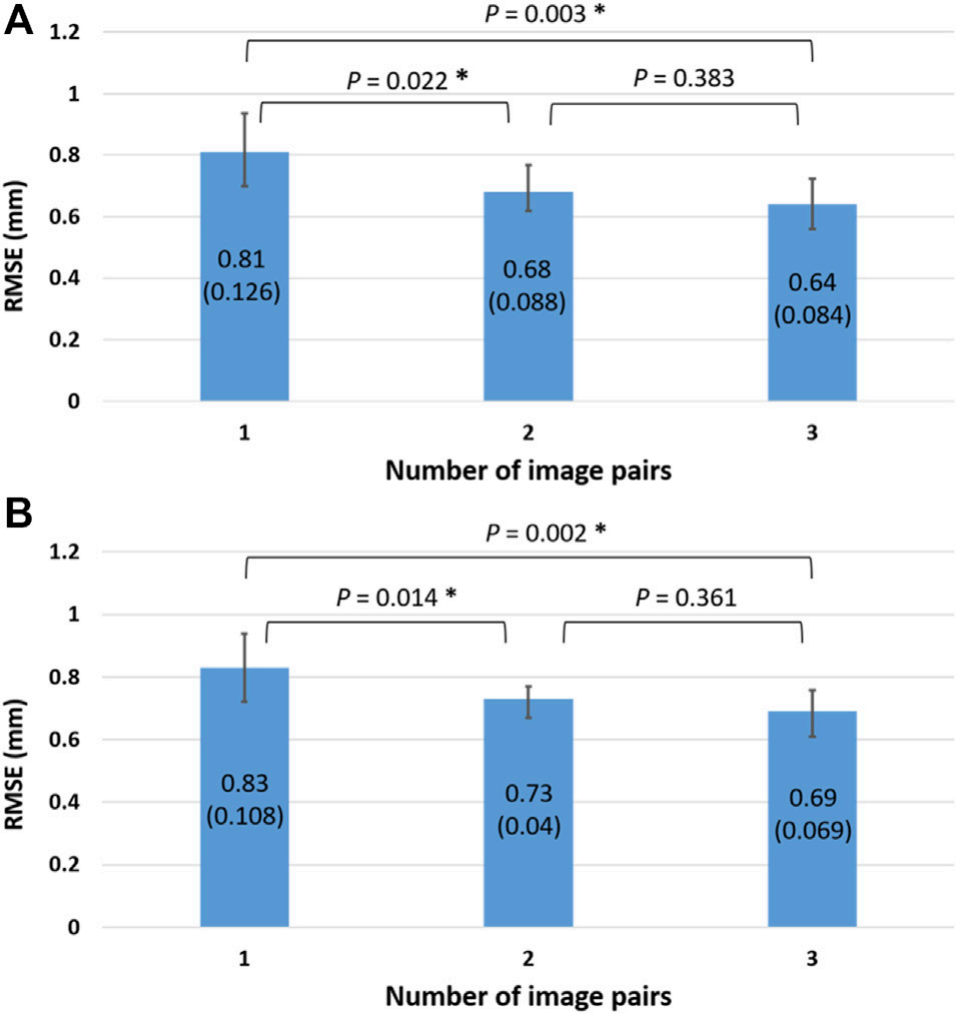
The means (standard deviations) of the RMSE (unit: mm) for the *in vivo* femur **(A)** and tibia **(B)** shape reconstruction using 1, 2, and 3 image pairs. The asterisks indicate significant differences (*p* < 0.05).

The RMSE of the reconstructed knee models using one image pair for the femur and tibia were 0.81 ± 0.126 and 0.83 ± 0.108 mm, respectively, Figure 5). The corresponding values for two and three image pairs were 0.68 ± 0.088 and 0.73 ± 0.04 mm, and 0.64 ± 0.084 and 0.69 ± 0.069 mm, respectively, (Figure 5). The RMSE of both the femur and tibia for one image pair were significantly greater than those for two and three image pairs, while no significant differences existed between two and three image pairs (Figure 5). The RMSE of the reconstructed knee models were significantly reduced after Phase 2 optimization for all image pair conditions for the tibia and for 1, 2, and image pairs for the femur when compared to those with only Phase 1 optimization (Table 2). Comparisons of these results with those of previous studies are also shown in Table 3.

**TABLE 2:** Means (standard deviations) of the RMSE (unit: mm) for the *in vivo* femur and tibia shape reconstruction after Phase 1 and Phase 2 using the two-phase optimization method.

Model	No. of image pairs	Phase 1	Phase 2	p
Femur	1 pair	0.89 (0.12)	0.81 (0.13)	< 0.05^*^
	2 pairs	0.79 (0.11)	0.68 (0.09)	< 0.05^*^
	3 pairs	0.79 (0.08)	0.64 (0.08)	< 0.05^*^
Tibia	1 pair	1.06 (0.19)	0.83 (0.11)	< 0.05^*^
	2 pairs	0.91 (0.08)	0.73 (0.04)	< 0.05^*^
	3 pairs	0.83 (0.10)	0.69 (0.07)	< 0.05^*^

*: *p*< 0.05, significant difference.

**TABLE 3:** Comparisons of the bone type, number of reconstructed models, number of fluoroscopy images, methods of modeling (number of PCA if using SSM), absolute mean errors, RMSE, image synchronization, and the experiment type for 3D knee shape reconstruction between published studies and the current study.

	Bone	No. of models tested	No. of images	Method (No. of PCA)	Absolute mean error (mm)	RMSE (mm)	Fluoroscopy images	Experiment
Fleute *et al.* Fleute and Lavallée (1999)	Distal femur	**-**	2	SSM (-)	**-**	0.99–1.33	synchronized	simulation
Laporte *et al.* Laporte et al. (2003)	Distal femur	8	2	NSCC (-)	-	1.4	synchronized	*in vitro*
Tang and Ellis. Tang and Ellis (2005)	Proximal/Distal femur	2	3	Statistical Atlas (9/7)	-	1.72/1.95	synchronized	simulation
Filippi *et al.* Filippi et al. (2008)	Full femur	5	2	FFD (-)	1.4	-	synchronized	simulation
Gamage *et al.* Gamage et al. (2009)	Full femur	6	2	Generic model (-)	0.86	-	synchronized	*in vitro*
Zhu and Li. Zhu and Li (2011)	Distal femur	10	2	SSM (-)	0.9	-	synchronized	*in vitro*
Baka *et al.* Baka et al. (2011)	Distal femur	10	2	SSM (30)	**-**	1.68	synchronized	*in vitro*
Karade *et al.* Karade and Ravi (2015)	Distal femur	5	2	LSD (-)	1.2	1.4	synchronized	*in vitro*
Tsai *et al.* Tsai et al. (2015)	Distal femur/Proximal tibia	4	2	SSM (20/30)	0.67/0.45	-	synchronized	*in vitro*
Yu et al. Yu et al. (2016)	Proximal femur	10	2	SSM (-)	1.29	-	synchronized	simulation
Valenti et al. Valenti et al. (2016a)	Distal femur	40	6	SSM (-)	0.43–2.19	-	synchronized	simulation
Valenti et al. Valenti et al. (2016b)	Distal femur	1	6	SSM (-)	Median errors: 3–4 mm	-	synchronized	simulation
van IJsseldijk et al. van IJsseldijk et al. (2016)	Distal femur/Proximal tibia	6	1	SSM (-)	-	0.49–0.74	single plane	*in vivo*
Cerveri et al. Cerveri et al. (2017)	distal femur	6	2	SSM (-)	-	0.7–0.8	-	simulation
Smoger *et al.* Smoger et al. (2017)	Patella	40	10	SSM (-)	0.45	-	synchronized	simulation
Yu et al. Yu et al. (2017)	Proximal femur	20	2	SSM (-)	1.3	-	synchronized	simulation
Zheng et al. Zheng and Yu (2017)	Femur	10	2	SSM (-)	1.3	-	synchronized	simulation
Fotsin et al. Fotsin et al. (2019)	Femur/Tibia/Fibula	18	2	SSM (-)	-	0.72/0.99/0.82	-	simulation
Cerveri et al. Cerveri et al. (2020)	Distal femur+	99	-	SSM (-)	1.28	-	-	simulation
	Proximal tibia	5	1	SSM (-)	-		single plane	*in vivo*
Wu et al. Wu and Mahfouz (2021)	Femur/Tibia					1.19/1.15		
This study	Distal femur/Proximal tibia	10	2	SSM (20/20)	0.61/0.68	0.81/0.83	asynchronized	*in vivo*
			4		0.58/0.60	0.68/0.73		
			6		0.52/0.55	0.64/0.69		

## Discussion

The current study aimed to develop a new two-phase optimization approach for SSM-based 3D subject-specific knee model reconstructions using more than one asynchronous fluoroscopy image pair from widely available alternating bi-plane fluoroscopy systems in clinical settings. The approach was implemented and evaluated using a CT-based SSM model of the knee for a healthy young Chinese male population. Both computer simulation and *in vivo* evaluations shoCw that the new two-phase optimization approach with SSM was capable of reconstructing subject-specific knee models with high accuracy, and two or three image pairs achieved a much better accuracy than using a single image pair for both the femur and tibia. It was also found the two-phase optimization was indeed producing more accurate results than single optimization phases. The performance of the SSM model of the knee was affected by several factors, primarily the population and number of training models, shape correspondence, shape alignment, and the selection of the principal components (Baka et al., 2011; Sarkalkan et al., 2014; Tsai et al., 2015). The number of training models in the current study was higher than that of most previous studies (from 22 to 43 training models) (Baka et al., 2011; Zhu and Li, 2011; Karade and Ravi, 2015), covering greater variability of the geometric features of the joint. The current SSM of the knee joint based on a healthy young Chinese male population has been shown to produce results with high reconstruction accuracy for other subjects in the same population. This may be expected to be better than using Caucasian-based SSM because ethnic differences in knee morphology have been observed (Baka et al., 2011; Baka et al., 2012; Karade and Ravi, 2015; Tsai et al., 2015). However, further studies will be needed to confirm whether the current Chinese-based SSM would enable subject-specific reconstruction of a Caucasian knee at the same accuracy. Further inclusion of models with a wider range of sizes and morphological features will also be needed for broader applications. In the current approach, the entire pre-processing for mesh correspondence between training models was fully automated and free from any manual interventions, avoiding the possible variability in node distributions resulting from manual digitization (Zhu and Li, 2011; Tsai et al., 2015), and thus contributing to the final accuracy.

The shape modes used for representing subject-specific models were chosen considering the accuracy and computational efficiency. The first few principal components with higher eigenvalues contributed to the major shape variances, but the number of shape modes chosen may vary among SSM models depending on the population or ethnic group from which the training datasets were obtained. For example, the femur was determined with the first 20, while the tibia was determined with the first 30 by Tsai et al. (2015); Baka *et al.* adopted the first 30 PCA to retain 95% of the variance (Baka et al., 2011); while the first nine and first seven modes were chosen for the proximal and distal femur to account for accumulated shape variances of about 85.9 and 86.1%, respectively, by Tang *et al.* (Tang and Ellis, 2005). These previous SSM models were all based on Caucasian groups or a Caucasian population. In the current SSM model for the Chinese population, accumulated shape variances of about 90% were achieved with the first 20 PCA for both the femur and tibia. While the more the number of shape parameters used, the higher the accuracy a training model can be described, the choice of the number of shape parameters can have direct impact on the accuracy and efficiency of the reconstruction of a subject-specific 3D knee model from planar images.

The new two-phase optimization approach has several important features to achieve high accuracy (sub-millimeter in RMSE) and efficiency in 3D subject-specific reconstruction of the knee. Firstly, the 2-phase optimization scheme used model-generated DRRs for the 3D/2D image registration in the optimization procedure, contributing to the observed high accuracy in reconstructing subject-specific knee models (Table 1; Figure 5). This is in contrast to previous methods using mainly the contours of the model projected onto the fluoroscopy imaging plane (Laporte et al., 2003; Baka et al., 2011; Zhu and Li, 2011; Baka et al., 2014; Karade and Ravi, 2015). While the assumed homogeneous density of the bone model did not reproduce the real CT radiodensity information, the resulting attenuation of bony contours and structural overlapping on the DRRs (e.g., bilateral condyles) helped improve the structural similarities with the fluoroscopy images. Secondly, by taking the shape and bone pose parameters as design variables and the summation of the similarity measures of all the image pairs as objective function, the new 2-phase optimization approach was successful in tackling the problems of temporal and spatial asynchronization of the bones (changes in bone poses) involved in the imaging at different time instances in the reconstruction of subject-specific bone models. Using two or three pairs of fluoroscopy images from a clinical alternating bi-plane fluoroscopy system, very high accuracy with an RMSE of less than 0.74 mm was achieved for both femur and tibia. This is better than most previously reported SSM-based methods using a single synchronized image pair for the femur with RMSE ranging from 1.33 to 1.68 mm (Fleute and Lavallée, 1999; Laporte et al., 2003; Baka et al., 2011; Karade and Ravi, 2015) (Table 3). Baka et al. (2014) used multiple frames of images of the knee during particular tasks from two fixed perspective views of a synchronous bi-plane fluoroscopy system. The shape customization process would thereby involve simultaneously searching for the optimal shape parameters and the pose parameters from a great number of image frames. In contrast, the current approach acquired fluoroscopy image pairs from 4 different views (0°, 30°, 45°, and 60°) with respect to the subject’s knee by rotating the subject with a custom-made rotating plate so the x-ray image pairs from various perspectives could capture more skeletal features (Figure 3). Also, the knee images were collected in the standing posture instead of during motion tasks as in Baka et al. for images with less motion blur. The transformations among the fluoroscopic views were determined during an experimental calibration procedure, so the shape customization process involved fewer unknown parameters (i.e., shape parameters and pose parameters in one instant). All these features were considered beneficial to the shape reconstruction process. Thirdly, by taking only the first 10 shape parameters with an accumulated variance of 85% in Phase 1, the new approach enabled a relatively fast search of a first estimate of the shape and pose of the bone. This was then followed by the refined search with the second 10 shape parameters with an accumulated variance of 90% in Phase 2 for a significantly increased accuracy (Table 2). With these new features, the 2-phase optimization approach was able to produce results with sub-millimeter accuracy in the 3D knee shape reconstruction as compared to previous studies (Table 3).

The current computer simulations showed that more synchronized image pairs improve reconstruction performance. Similar outcomes were also found in previous computer simulation studies (Tang and Ellis, 2005). However, under real life conditions, the addition of an extra pair of images did not increase the reconstruction accuracy as shown by the current *in vivo* study (Tables 1), presumably owing to the errors arising from the temporal and spatial asynchronization of the images. The current results suggest that the reconstruction accuracy could benefit from more images as long as the temporal and spatial asynchronization of these images are taken into account in the reconstruction procedure. Considering both reconstruction quality and computing efficiency, two image pairs would be the best choice for subject-specific model reconstruction when using clinically obtained asynchronous images.

The current study established the first SSM model of the knee for a healthy young Chinese male population. With the proposed 2-phase optimization approach, the reconstruction of subject-specific knee model from two or more pairs of temporally and spatially asynchronous fluoroscopy images was made possible, and was shown to produce highly accurate results. Further inclusion of other types of knee models, such as females and older people and those with disease or deformities, in the training database would be helpful for expanding the current database for future basic research and clinical applications such as studies of the features of specific diseases or deformities of the joint. Further inclusion of Caucasian knee models in the training database or as test models would also enable quantitative comparisons of the performance of the new approach in subject-specific model reconstructions for Chinese and Caucasian subjects using different datasets. With the accuracy of the SSM model and the 2-phase reconstruction method, the proposed approach will also be useful for studying image-based knee kinematics during functional activities, as well as for clinical applications using asynchronous fluoroscopy systems.

## Conclusion

A new two-phase optimization approach was developed for SSM-based 3D subject-specific knee model reconstructions using more than one asynchronous fluoroscopy image pair from widely available alternating bi-plane fluoroscopy systems in clinical settings. A CT-based SSM model of the knee was also developed for a healthy young Chinese male population. Both computer simulation and *in vivo* evaluations show that the new optimization approach was capable of reconstructing subject-specific knee models with high accuracy, and two or three image pairs achieved a much better accuracy than using a single image pair for both the femur and tibia. Considering computational costs, two image pairs may be preferred over three image pairs. The new approach will be useful for generating patient-specific knee models from SSM models for clinical applications using multiple asynchronous images from alternating bi-plane fluoroscopy widely available in clinical settings. The current SSM model will serve as a basis for further inclusion of training models with a wider range of sizes and morphological features for broader applications.

## Data Availability

The datasets used and/or analyzed during the current study are available from the corresponding author on reasonable request. Requests to access the datasets should be directed to T-WL, twlu@ntu.edu.tw.
